# An efficacy evaluation method for non-normal outcomes in randomized controlled trials

**DOI:** 10.1038/s41598-019-47727-y

**Published:** 2019-08-06

**Authors:** Yang Li, Zhang Zhang, Qian Feng, Danhui Yi, Fang Lu

**Affiliations:** 1 Center for Applied Statistics, Renmin University of China, Beijing, China; 2 School of Statistics, Renmin University of China, Beijing, China; 3 Statistical Consulting Center, Renmin University of China, Beijing, China; 4 Xiyuan Hospital, China Academy of Chinese Medical Sciences, Beijing, China; 5 School of Mathematics and Statistics, The University of Melbourne, Melbourne, Australia

**Keywords:** Drug development, Epidemiology

## Abstract

Randomized controlled trials (RCT) are widely used in clinical efficacy evaluation studies. Linear regression is a general method to evaluate treatment efficacy considering the existence of confounding variables. However, when residuals are not normally distributed, parameter estimation based on ordinary least squares (OLS) is inefficient. This study introduces an exponential squared loss (ESL) model to evaluate treatment effect. The proposed method provides robust estimation for non-normal data. Simulation results show that it outperforms ordinary least squares regression with contaminated data. In the mild cognitive impairment (MCI) efficacy evaluation study with traditional Chinese medicine, our method is applied to construct a linear efficacy evaluation model for the difference in Alzheimer’s disease assessment scale-cognitive (ADAS-cog) scores between the final and baseline records (ADASFA), with the existence of confounding factors and non- normal residuals. The results coincide with existing medical literatures. This proposed method overcomes the limitation of confounding variables and non-normal residuals in RCT efficacy studies. It outperforms OLS on estimation efficiency in situations where the percentage of non-normal contamination reaches 30%. These advantages make it a good method for real-world clinical studies.

## Introduction

Mild cognitive impairment (MCI) is a syndrome defined as a cognitive decline, which may affect daily activities. The amnesic subtype of MCI has a high risk of progression to Alzheimer’s disease and could lead to a prodromal stage of this disorder^1^. Alzheimer’s disease assessment scale-cognitive (ADAS-cog) subscale measures the progression of MCI in 11 relevant fields, namely spoken language ability, comprehension of spoken language, recall of test instructions, word-finding difficulty, following commands, naming, constructions, ideational praxis, orientation, word recall, and word recognition. Detailed information on ADAS-cog subscale can be found in Rosen *et al*.^2^.

Institute of Clinical Pharmacology at Xiyuan Hospital conducted a phase III randomized clinical trial to evaluate the efficacy of a traditional Chinese prescription on MCI. The double blinded randomized clinical trial was conducted in eight qualified medical centres across China with 216 patients allocated to the treatment arm and 108 retained as control. Two patients dropped out from each arm, resulting in 320 complete observations in the final dataset. Data on the difference between final ADAS-cog score and baseline scores (ADASFA) were recorded for the efficacy study. Previous literatures have used ADASFA in efficacy evaluation of MCI or Alzheimer’s disease^3,4^. The most intuitive idea is to test whether the treatment and control means are equal. However, Morgan and Rubin^5^ argued that the baseline equivalence is not guaranteed although the allocation is randomized. Imbalance in baseline covariates could confound the statistical test when comparing ADASFA between the two arms. Ten variables, specifically age, height, weight, gender, education, ethnicity, occupation, centre, drug (whether the patient took a drug for MCI in the past three months), and ADAS1 (the baseline record of ADAS-cog^6^), were recorded as potential covariates. Table 1 shows descriptive statistics of these variables. The explorative covariance analysis presented in Table 2 indicates that the variable of centre and ADAS1 may confound the efficacy evaluation of ADASFA. This implies that a linear regression model should be involved rather than using a simple statistical test in this study, that is,

$$y={\beta }_{0}+{\beta }_{1}{x}_{1}+{\beta }_{2}{x}_{2}+\cdots +{\beta }_{p}{x}_{p}+\varepsilon $$

where *p* is the number of covariates. We denote a binary variable *x*_*j*_ = 1 to represent the treatment arm and *x*_*j*_ = 0 for the control arm. The efficacy can be evaluated by the corresponding coefficient *β*_*j*_ ^7^. Ordinary least squares (OLS) is a general parameter estimation method for simple linear regression which performs as the best linear unbiased estimation when assuming independent identical normally distributed errors:

$$\varepsilon |x \sim N(0,{\sigma }^{2}).$$

**Table 1:** Descriptive statistics of variables.

Discrete	Categories	Sample size		Continuous	Mean ± SD		Median	
		Treatment arm	Control arm		Treatment arm	Control arm	Treatment arm	Control arm
gender	male	88	46	age	62.75 ± 7.90	63.77 ± 8.28	62.00	62.50
	female	126	60	height (cm)	163.80 ± 7.27	164.43 ± 7.45	163.00	163.00
education	primary	40	26	weight (kg)	64.34 ± 9.23	65.09 ± 9.46	65.00	65.00
	middle and above	174	80	ADAS1	14.83 ± 6.40	15.11 ± 6.12	13.85	14.85
ethnicity	Han	206	105	**ADASFA**	**3.99** ± **4.11**	**4.13** ± **3.92**	**3.67**	**4.00**
	Non-Han	8	1					
occupation	Physical	55	31					
	Mental	159	75					
drug	without	155	71					
	with	59	35					
centre	centre1	23	10					
	centre2	8	4					
	centre3	32	16					
	centre4	24	12					
	centre5	24	12					
	centre6	48	24					
	centre7	24	12					
	centre8	31	16					
**Total**		**214**	**106**

**Table 2:** An example of covariance analysis.

	Degrees of freedom	Sum of squares	Mean square	F-value	P-value
group	1	3.43	3.43	0.31	0.58
centre	7	375.73	53.68	4.80	<0.001
group*centre	7	104.41	14.92	1.33	0.234
ADAS1	1	1439.67	1439.67	128.79	<0.001
error	303	3387.00	11.18		
Corrected total	319	5204.11			

**Figure 1:**
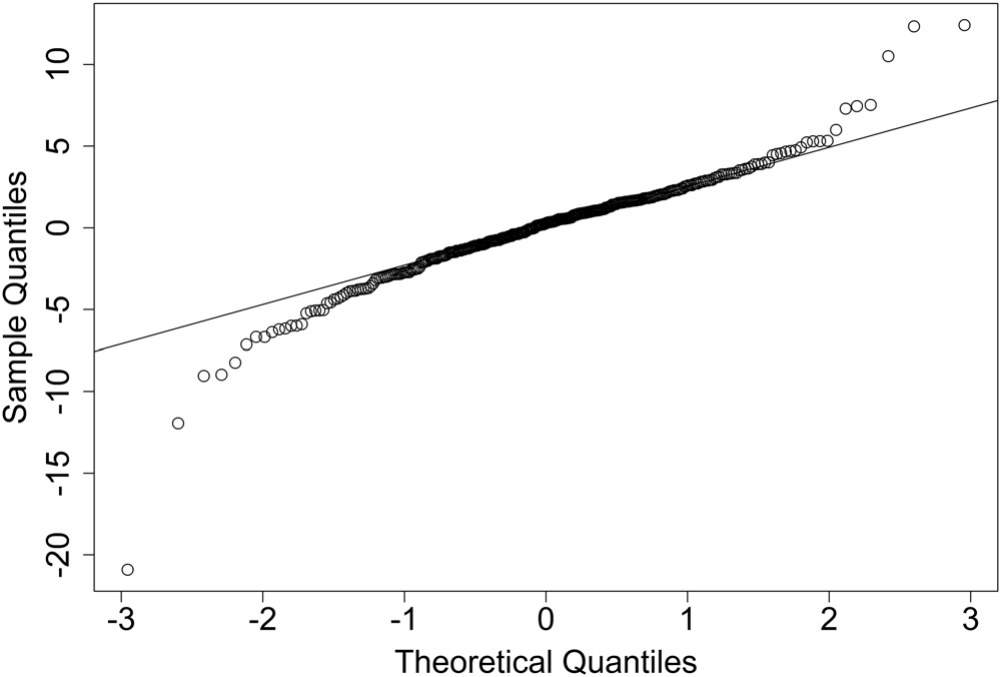
QQ plot of residuals in the MCI study using OLS.

However, the QQ plot in Fig. 1 shows that the MCI dataset may not follow a normal distribution and a Shapiro-Wilk test (W=0.9283, p-value = 2.799e-11) also suggests a similar result. The contaminated non-normal part may come from either measurement error or mixed distribution^8^, which is commonly presented in medical studies^9,10^. This could lead to inefficient efficacy estimation by using OLS since the contaminated part is not addressed^11^. A more robust estimation method in linear regression is, therefore, required in such studies.

Many robust methods have been discussed in literatures. Bao^12^ developed a rank-based estimate in linear regression. Wang *et al*.^13^ proposed an robust estimation via least absolute deviation while Wang *et al*.^14^ introduced an exponential squared loss (ESL) to select variables robustly. Since the breakdown point of ESL is almost 50%, we adopt it in the MCI efficacy evaluation study. Numerical studies show that the proposed method can achieve a more accurate estimation with a large proportion of contamination in the dataset. Additionally, the estimations are consistent with OLS when contamination proportions are relatively low. Therefore, it can be used as a complementary efficacy evaluation method in real-world clinical studies regardless of the presence or lack of contaminations.

## Methods

### Model

Suppose there are *n* subjects, denoted as ${\{({x}_{i},{y}_{i})\}}_{i=1}^{n}$ where *y*_*i*_ is the outcome and *x*_*i*_ = (*x*_*i*1_, …, *x*_*ip*_)^*T*^ is a *p*-dimensional vector of covariates. A linear regression model is,

$${y}_{i}={x}_{i}^{T}\beta +{\varepsilon }_{i},i=1,2,\,\cdots ,\,n$$

where *β* is a *p*-dimensional vector of unknown parameters while *ε*_*i*_ is independent and identically distributed with some unknown distribution satisfying *E*(*ε*_*i*_) = 0 and *ε*_*i*_ ╨ *x*_*i*_.

The ESL function has been used in AdaBoost for classification problems with success^15^. Wang *et al*.^14^ expanded the use of the ESL function for robust variables selection. We now use it to estimate parameters in linear regression without sparsity. The ESL function is defined as

$${{\rm{\Phi }}}_{\gamma }(t)=1-\exp (-\,\frac{{t}^{2}}{\gamma }),$$

which is a function of *t*, and *γ*, where the latter is a tuning parameter. To estimate model parameters (*β*), the objective function of ESL is to maximize,

1 $${l}_{n}(\beta )=\sum _{i=1}^{n}\,\exp (-\,\frac{{({y}_{i}-{x}_{i}^{T}\beta )}^{2}}{\gamma }).$$

The tuning parameter *γ* controls the degree of robustness of the estimator. With a relatively large *γ*, the proposed estimator gets close to the OLS estimator while a smaller *γ* leads to a limited influence of contaminations on the estimator. Since the tuning parameter *γ* controls the degree of robustness and efficiency of the estimator, a data-driven procedure that yields both high robustness and high efficiency simultaneously is used to select an appropriate *γ*. The entire calculation process in terms of ESL borrows from the idea proposed in Wang *et al*.^14^:

1. **Find the pseudo outlier set of the sample**. Let *D*_*n*_ = {(*x*_1_, *y*_1_), …, (*x*_*n*_, *y*_*n*_)}. Then, calculate ${r}_{i}({\hat{\beta }}_{n})={y}_{i}-{x}_{i}^{T}{\hat{\beta }}_{n},\,i=1,2,\,\cdots ,\,n$ and ${S}_{n}=1.4826\times {{\rm{median}}}_{i}|{r}_{i}({\hat{\beta }}_{n})-{{\rm{median}}}_{j}({r}_{j}({\hat{\beta }}_{n}))|$. Take the pseudo outlier set as ${D}_{m}=\{({x}_{i},{y}_{i}):{r}_{i}({\hat{\beta }}_{n})\ge 2.5{S}_{n}\}$, where *m* is the cardinality of *D*_*m*_ set and *D*_*n*−*m*_ = *D*_*n*_/*D*_*m*_.
2. **Update the tuning parameter** ***γ***. Let *γ* be the minimiser of det $(\hat{V}(\gamma ))$ in the set *G* = {*γ*:*ζ*(*γ*) ∈ (0, 1]}, and $\zeta (\gamma )=2m/n+(2/n)\sum _{i=m+1}^{n}\,{{\rm{\Phi }}}_{\gamma }{r}_{i}({\hat{\beta }}_{n}),\hat{V}(\gamma )={\{{\hat{I}}_{1}({\hat{\beta }}_{n})\}}^{-1}{\rm{\Sigma }}{\{{\hat{I}}_{1}({\hat{\beta }}_{n})\}}^{-1}$, det (⋅) denotes the determinant operator, and
  $$\begin{array}{rcl}{\hat{I}}_{1}({\hat{\beta }}_{n}) & = & \frac{2}{\gamma }\{\frac{1}{n}\sum _{i=1}^{n}\,\exp (\,-\,{r}_{i}^{2}({\hat{\beta }}_{n})/\gamma )\}(\frac{2{r}_{i}^{2}({\hat{\beta }}_{n})}{\gamma }-1)\}\times (\frac{1}{n}\sum _{i=1}^{n}\,{x}_{i}{x}_{i}^{T})\\ \hat{{\rm{\Sigma }}} & = & cov\{\exp (\,-\,{r}_{i}^{2}({\hat{\beta }}_{n})/\gamma )\frac{2{r}_{i}^{2}({\hat{\beta }}_{n})}{\gamma }{x}_{i}\times \cdots \times \exp (\,-\,{r}_{n}^{2}({\hat{\beta }}_{n})/\gamma )\frac{2{r}_{i}^{2}({\hat{\beta }}_{n})}{\gamma }{x}_{n}\}\end{array}$$
3. **Update** ${\hat{{\boldsymbol{\beta }}}}_{{\boldsymbol{n}}}$. After selecting *γ* in step 2, update ${\hat{\beta }}_{n}$ by maximizing (1).

We set the MM estimator^16^ ${\tilde{\beta }}_{n}$ as the initial estimator. The algorithm is an iterative procedure as shown above. To attain high efficiency, we choose the tuning parameter *γ* by minimizing the determinant of asymptotic covariance matrix as in Step 2. Since the calculation of det $(\hat{V}(\gamma ))$ depends on the estimation of ${\tilde{\beta }}_{n}$, we update ${\tilde{\beta }}_{n}$ in Step 3 and repeat the algorithm until the convergence condition $\Vert {\hat{\beta }}_{n}^{old}-{\hat{\beta }}_{n}^{new}\Vert  < {10}^{-2}$ is satisfied.

### Simulations

In order to verify the performance of the introduced method, we conduct numerical studies to compare bias and mean squared errors (MSE) of the estimators of our algorithm (ESL) versus those from the ordinary least squares (OLS).

Simulate data ${\{({x}_{i},{y}_{i})\}}_{i=1}^{n}$ as follows, where *x*_*i*_ = (*x*_*i*1_, *x*_*i*2_, …, *x*_*ip*_)^*T*^, *i* = 1, 2, …, *n* with *p* = 7 and *n* = 300. The first six covariates are continuous, that is, *x*_*ij*_ ~ *N*(0, 1) for *j* = 1, 2, …, 6 and *x*_*i*7_ is categorical, selected from {1, 2, …, 4}. Convert *x*_*i*7_ into three binary variables, denoted as *z*_*i*1_, *z*_*i*2_, *z*_*i*3_ where *z*_*ij*_ represents whether *x*_*i*7_ belongs to the *j*-th category and *z*_*i*1_ = *z*_*i*2_ = *z*_*i*3_ = 0 means *x*_*i*7_ belongs to the last category. Thus, we have *x*_*i*_ = (*x*_*i*1_, *x*_*i*2_, …, *x*_*i*6_, *z*_*i*1_, *z*_*i*2_, *z*_*i*3_)^*T*^. Let *β* = (*β*_0_, *β*_1_, …, *β*_9_)^*T*^ where *β* = (1, 1.2, 1.4, 1.6, 1.8, 2, 2.2, 2.4, 2.6, 2.8)^*T*^. The error term of contamination (outlier) follows *t*(1) distribution, and the error term of non-outlier follows standard normal distribution, *N*(0, 1). The proportion of contamination considered is 10%, 20% and 30%, respectively. For each proportion of contamination, the average mean, bias, standard deviation (SD), and MSE of ESL and OLS over 100 replications is reported in Table 3.

**Table 3:** Average results over 100 replications of ESL and OLS for 10%, 20%, and 30% contamination proportions, respectively.

Contamination Proportion (%)	_*β*_	ESL				OLS			
		Mean	Bias	SD	MSE	Mean	Bias	SD	MSE
10	1.000	1.005	0.005	0.120	0.014	1.000	0.000	1.199	1.424
	1.200	1.200	0.000	0.059	0.003	0.757	−0.443	4.717	22.225
	1.400	1.388	−0.012	0.067	0.005	1.206	−0.194	0.983	0.994
	1.600	1.608	0.008	0.062	0.004	1.111	−0.489	4.940	24.404
	1.800	1.788	−0.012	0.063	0.004	2.283	0.483	4.944	24.430
	2.000	2.000	0.000	0.067	0.004	1.797	−0.203	2.451	5.989
	2.200	2.200	0.000	0.073	0.005	2.576	0.376	3.423	11.739
	2.400	2.413	0.013	0.196	0.038	2.533	0.133	2.635	6.893
	2.600	2.613	0.013	0.172	0.029	2.209	−0.391	2.848	8.183
	2.800	2.761	−0.039	0.166	0.029	4.040	1.240	12.925	166.923
20	1.000	0.998	−0.002	0.119	0.014	0.952	−0.048	1.180	1.380
	1.200	1.206	0.006	0.057	0.003	1.824	0.624	6.962	48.375
	1.400	1.399	−0.001	0.061	0.004	2.094	0.694	4.178	17.763
	1.600	1.594	−0.006	0.052	0.003	1.389	−0.211	4.044	16.235
	1.800	1.800	0.000	0.072	0.005	3.146	1.346	9.641	93.837
	2.000	2.001	0.001	0.058	0.003	2.802	0.802	9.554	91.002
	2.200	2.215	0.015	0.064	0.004	1.144	−1.056	12.393	153.157
	2.400	2.388	−0.012	0.190	0.036	2.768	0.368	4.622	21.282
	2.600	2.621	0.021	0.169	0.029	−0.159	−2.759	36.200	1304.968
	2.800	2.783	−0.017	0.157	0.025	2.750	−0.050	2.829	7.927
30	1.000	1.001	−0.199	0.153	0.090	0.490	−0.710	4.589	21.518
	1.200	1.210	−0.590	0.071	0.380	4.637	2.837	41.919	1759.396
	1.400	1.394	−1.006	0.072	1.044	0.477	−1.923	11.368	132.534
	1.600	1.608	−0.059	0.071	0.664	2.416	0.749	9.555	91.850
	1.800	1.805	0.205	0.077	0.074	2.129	0.529	3.359	11.552
	2.000	1.996	−0.204	0.073	0.074	−0.272	−2.472	25.998	679.801
	2.200	2.201	0.067	0.075	0.662	5.254	3.121	39.104	1536.506
	2.400	2.389	0.989	0.204	1.047	−6.055	−7.455	106.666	11395.273
	2.600	2.607	0.607	0.221	0.444	3.612	1.612	5.673	34.700
	2.800	2.808	0.208	0.215	0.116	5.459	2.859	17.469	312.362

**Figure 2:**
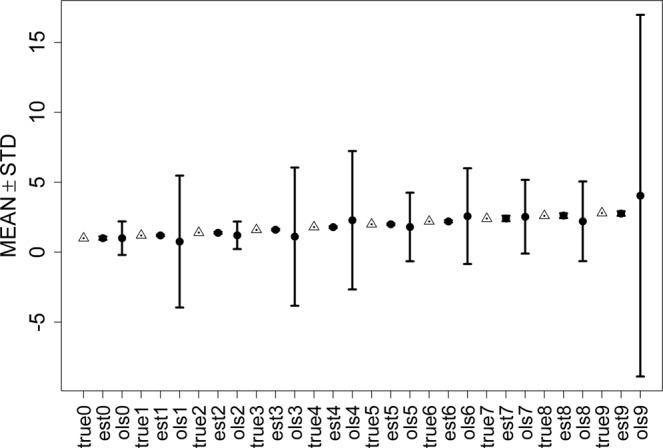
Error bars of ESL and OLS with 10% contamination.

**Figure 3:**
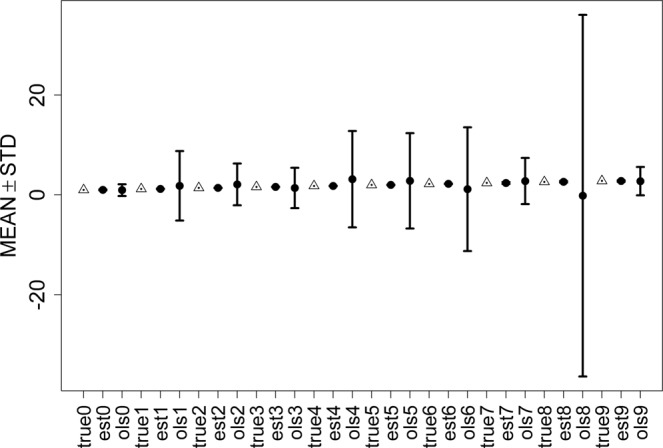
Error bars of ESL and OLS with 20% contamination.

**Figure 4:**
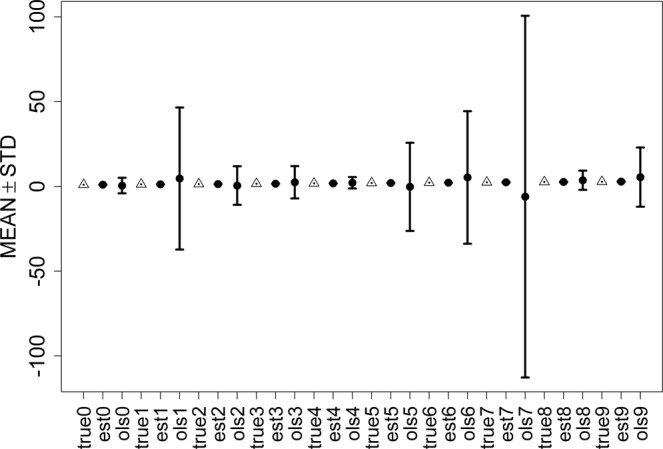
Error bars of ESL and OLS with 30% contamination.

Figure 2–4 show error bars of ESL and OLS with three proportions of contaminations, where the triangular points represent true values of parameters, the circles points represent means of estimator means,s and vertical lines mean represent standard deviations. ‘true*j*’, ‘esl*j*’, and ‘ols*j*’ refer to the corresponding parameter j for true value, ELS, and OLS estimations. It can be seen that the widths of error bars using ESL are significantly considerably shorter than those using OLS, which implies that the standard deviation of the ESL estimator in ESL is much smaller than that in of OLS, and our method is more robust.

## Results

In the MCI study, the linear regression model is conducted as follows:

$$\begin{array}{rcl}ADASFA & = & {\beta }_{1}age+{\beta }_{2}bmi+{\beta }_{3}ADAS1+{\beta }_{5}centre2+{\beta }_{6}centre3\\  &  & +\,{\beta }_{7}centre4+{\beta }_{8}centre5+{\beta }_{9}centre6+{\beta }_{10}centre7+{\beta }_{11}group\\  &  & +\,{\beta }_{12}gender+{\beta }_{13}education+{\beta }_{14}occupation+{\beta }_{15}drug+\varepsilon \end{array}$$

We include these variables in the model based on our clinical experience and existing literatures. In addition, we transform weight and height into a new variable BMI, since there are discussions on whether BMI has an effect on MCI. We exclude ethnicity and marital status from our model mainly because these two variables are extremely unbalanced between the treatment and control arms, and also due to the fact that almost no literature suggests that these two variables have an effect on MCI. Since 11 out of 16 variables are categorical variables, we do not consider interaction effects. The by-centre descriptive analysis is presented in Table 4 and 5.

**Table 4:** Continuous variable descriptive statistics.

Centre	Continuous	Treatment		Control		Treatment	Control
		Mean	Sd	Mean	Sd	Median	Median
1	age	56.39	7.48	58.90	8.37	53.00	58.00
	bmi	23.57	1.67	23.44	1.72	23.92	23.13
	ADAS1	12.36	4.99	12.03	6.91	13.00	12.70
	ADASCHA	2.60	2.00	2.18	2.11	2.40	2.00
2	age	66.00	7.60	66.75	8.30	67.50	67.50
	bmi	23.82	1.33	22.71	3.62	23.84	22.70
	ADAS1	8.08	3.78	8.25	3.52	7.30	8.00
	ADASCHA	2.63	3.19	4.68	3.30	2.55	5.00
3	age	64.25	6.41	62.63	7.39	64.00	61.00
	bmi	24.00	2.60	24.29	2.47	24.24	23.94
	ADAS1	17.70	5.57	18.53	5.63	17.00	19.15
	ADASCHA	5.68	3.45	4.86	4.16	4.87	4.55
4	age	64.88	7.92	64.50	9.02	66.50	63.50
	bmi	24.68	2.53	24.85	2.66	24.97	24.37
	ADAS1	20.71	6.49	21.11	5.76	19.65	20.85
	ADASCHA	4.22	3.31	6.55	6.12	4.14	5.55
5	age	65.71	9.07	60.75	6.77	66.00	59.50
	bmi	24.16	2.50	25.64	2.62	24.49	26.02
	ADAS1	18.39	5.52	17.91	5.13	19.33	19.35
	ADASCHA	4.53	3.63	4.91	1.43	4.37	5.05
6	age	64.63	7.55	67.83	6.55	66.00	69.00
	bmi	24.55	3.27	23.84	3.09	24.57	23.80
	ADAS1	10.55	4.75	10.87	3.37	9.40	10.30
	ADASCHA	3.94	4.46	4.59	3.33	3.64	4.00
7	age	58.17	5.95	57.42	4.87	57.50	56.00
	bmi	22.69	1.99	22.96	2.80	22.99	22.23
	ADAS1	15.69	5.84	14.66	3.60	14.85	14.95
	ADASCHA	3.21	6.69	2.78	5.06	4.70	3.80
8	age	61.81	6.96	67.63	9.54	60.00	70.50
	bmi	23.45	3.48	23.77	3.59	22.66	23.14
	ADAS1	14.09	5.07	15.41	5.77	15.30	14.55
	ADASCHA	3.67	3.49	2.41	2.70	2.60	1.45

**Table 5:** Discrete variable descriptive statistics.

Centre	Group	Gender		Education		Ethnicity		Occupation		Drug	
		Male	Female	Primary	Middle and above	Han	Non-han	Physical	Mental	Without	With
1	Treatment	8	15	4	19	23	0	7	16	22	1
	Control	3	7	3	7	10	0	3	7	9	1
2	Treatment	2	6	1	7	8	0	1	7	4	4
	Control	4	0	1	3	4	0	0	4	0	4
3	Treatment	11	21	2	30	32	0	3	29	22	10
	Control	7	9	3	13	16	0	2	14	12	4
[0]*4	Treatment	15	9	6	18	23	1	9	15	18	6
	Control	6	6	4	8	12	0	5	7	9	3
5	Treatment	10	14	3	21	23	1	7	17	14	10
	Control	3	9	2	10	12	0	3	9	9	3
6	Treatment	22	26	9	39	43	5	16	32	30	18
	Control	13	11	10	14	23	1	10	14	13	11
7	Treatment	8	16	5	19	23	1	3	21	22	2
	Control	4	8	0	12	12	0	3	9	10	2
8	Treatment	12	19	10	21	31	0	9	22	23	8
	Control	6	10	3	13	16	0	5	11	9	7

Table 6 shows the parameter estimations using ESL and OLS. The empirical 95% confidence interval is calculated by the bootstrap approach. When the bootstrap confidence interval does not include 0, it indicates that the corresponding covariate has a significant effect on the primary outcome. Note that there are some differences between the ESL and OLS estimations. For example, the effects of centre5 and centre8 on ADASFA are opposite. Given the non-normal residuals, the ESL estimators are more accurate. From the results, we can conclude that

1. ADAS1 and centre 6 have significant influences on ADASFA since their bootstrap confidence intervals do not contain 0. From the medical view, higher ADAS1 means patients are in worse health situation, which can have a positive effect on ADASFA.
2. ESL and OLS both show that ADAS1 has a positive effect on decreasing ADAS-cog. For age, ESL shows that age has no effect on decreasing ADAS-cog because its bootstrap confidence interval contains 0 while OLS shows that age has a negative effect on decreasing ADAS-cog. From a medical viewpoint^17^, it is verified that ‘age’ has a significant effect on MCI. Prior work has demonstrated that rates of dementia increase exponentially with age^18,19^. However, the significant effect of age on MCI does not mean that it also influences the treatment effect.
3. The ESL group coefficient is −0.141 and its bootstrap confidence interval contains 0. This result makes sense because this project is a non-inferiority trial and the treatment group was not worse than the control group.
4. The ESL shows that centres 3, 6, and 7 have significant effects on the outcome. However, OLS shows that centres 3 and 7 have no significant impact but centre 6 has a significant effect on the outcome. According to Table 4, the average ADAS1 of centre 6 is much lower than that of centres 3 and 7, which implies that patients in centres 3 and 7 are in worse conditions. Moreover, patients in different centres may have different non-compliance levels, which may also contribute to the result that some centres have significant effects on the outcome while others do not.
5. Since we have shown that the data is not normally distributed, we can have greater confidence in the ESL results.

**Table 6:** Estimation results in MCI study using ESL and OLS.

Variables	ESL		OLS	
	Estimate	95% CI	Estimate	95% CI
intercept	0.241	(−4.412, 4.203)	−0.127	(−4.961, 4.554)
age	−0.05	(−0.098, 0.01)	−0.055	(−0.108, −0.003)
bmi	0.072	(−0.088, 0.22)	0.023	(−0.112, 0.158)
ADAS1	0.31	(0.221, 0.398)	0.434	(0.361, 0.508)
center2	1.82	(−0.331, 5.253)	3.011	(0.683, 5.336)
cente3	1.428	(0.127, 2.683)	0.725	(−0.848, 2.299)
center4	−0.524	(−2.192, 1.106)	−0.988	(−2.711, 0.733)
center5	0.553	(−0.604, 1.571)	−0.151	(−1.828, 1.526)
center6	2.396	(1.457, 3.308)	2.733	(1.263, 4.201)
center7	1.474	(0.451, 2.514)	−0.71	(−2.303, 0.889)
center8	−0.001	(−1.284, 1.407)	0.158	(−1.372, 1.688)
group(treatment = 1)	−0.141	(−0.8, 0.567)	−0.096	(−1.553, −0.022)
gender(female = 1)	−0.565	(−1.19, 0.275)	−0.786	(−0.118, 1.947)
education(midlle and above = 1)	0.383	(−0.441, 1.285)	0.921	(−1.275, 0.589)
occupation(mental = 1)	−0.245	(−0.991, 0.456)	−0.341	(−0.812, 0.892)
drug(with = 1)	−0.08	(−1.197, 0.912)	0.035	(−1.197, 0.912)

## Conclusion

In this paper, we discuss a method to evaluate efficacy in a randomized control MCI study. As many covariates may influence the outcome, a linear regression model is considered rather than comparing group means using t test or ANOVA. An exponential squared loss function, which is superior to OLS when dealing with non-normal residuals, is introduced in this study. Simulation results show that the ESL model yields more efficient estimation than OLS in non-normal data. The proposed method is also robust in the case of data with outliers. These advantages of the ESL model become more noticeable when the contamination percentage increases. The proposed method does not require the normal distribution assumption, offering new insight in the efficacy evaluation for practical researchers.
